# Extensive Collateral Venous Circulation and Anatomic Remodeling in Chronic Deep Vein Thrombosis

**DOI:** 10.1155/cric/8841986

**Published:** 2026-01-08

**Authors:** Demi Curbelo, Ian Lancaster, Alexander Yaylayan, Merrill Krolick

**Affiliations:** ^1^ HCA Healthcare/USF Morsani College of Medicine GME: Internal Medicine/Interventional Cardiology, HCA Florida Largo Hospital, Largo, Florida, USA

**Keywords:** collateral venous circulation, deep vein thrombosis, venography

## Abstract

Chronic deep venous thrombosis (DVT) can result in a significant venous outflow obstruction, often prompting the development of extensive compensatory mechanisms to maintain adequate circulation. We report a case of recurrent DVT, presenting with left lower extremity pain and edema. Venography revealed complete occlusion of the left common femoral vein with extensive collateral venous circulation. Given the presence of well‐formed collaterals and clinical stability, the patient was treated conservatively with continued anticoagulation without additional invasive intervention. This case highlights the physiologic adaptation of collateral formation that can allow for conservative management in chronic DVT and contributes to a better understanding of management strategies for high‐risk patients with recurrent thrombotic events.

## 1. Introduction

DVT occurs when a thrombus forms within the deep veins, most commonly in the lower extremities, obstructing normal blood flow. This process is influenced by Virchow′s triad, which identifies three key factors contributing to thrombus formation: endothelial injury, stasis of blood flow, and a hypercoagulable state. Endothelial damage, often caused by trauma or surgery, exposes the underlying tissues and activates the clotting cascade. Stasis, resulting from immobility, venous insufficiency, or other conditions like obesity, promotes the accumulation of clotting factors [1]. A hypercoagulable state, due to inherited conditions such as Factor V Leiden mutation, or acquired factors like malignancy or pregnancy, increases the likelihood of thrombosis [1]. These factors can lead to long‐term venous damage, resulting in chronic DVT and its associated complications, including post‐thrombotic syndrome (PTS) and recurrent thrombotic events [1].

Chronic DVTs are characterized by a prolonged venous obstruction, which often lead to the development of collateral venous circulation as a compensatory mechanism. In response to persistent thrombotic occlusion, the body undergoes angiogenesis, creating alternative venous pathways that allow for continued venous return, despite the obstruction of primary venous channels. These collateral vessels can significantly alleviate the symptoms of venous insufficiency and maintain limb function in the setting of long‐standing DVT. However, the extent and effectiveness of collateral circulation vary depending on several factors, including the location, duration of the thrombotic event and thrombus burden [2].

Venography remains a key diagnostic tool for assessing chronic DVT, offering detailed visualization of venous anatomy, the extent of occlusion, and the presence of collateral circulation. The identification of robust collateral circulation can significantly alter management strategies, as it may allow for conservative treatment with anticoagulation alone, bypassing the need for invasive interventions [3]. In cases where collateral pathways are insufficient or where there is significant venous obstruction, more invasive procedures, such as mechanical thrombectomy, angioplasty, or stenting, may be considered to restore patency and improve venous flow [4]. In our case report, we will discuss the case of a 43‐year‐old female with chronic lower extremity DVT with extensive collateralization reviewing the anatomic considerations and management strategies for chronic DVT.

## 2. Case Presentation

A 43‐year‐old female presented to the hospital with left lower extremity pain and edema. She noted a history of similar symptoms when she was diagnosed with DVT of her left lower extremity following significant trauma with pelvic fracture 3 years ago, which was treated with therapeutic enoxaparin followed by warfarin for approximately 6 months. One year later, she was found to have a recurrent left lower extremity DVT. Hypercoagulability workup demonstrated a Factor V Leiden mutation and she was subsequently started on long‐term anticoagulation with apixaban. She also reported family history of DVT. However, due to inconsistent anticoagulation use, she later developed an extensive DVT extending from her left iliac vein to the left popliteal vein and underwent mechanical thrombectomy with stent placement in the left iliac and femoral veins. She was counseled on the importance of medication adherence at that time. Five months prior to presentation, she was diagnosed with persistent, symptomatic left lower extremity DVT that was managed with repeat catheter‐based therapy in conjunction with continued anticoagulation.

**Figure 1:**
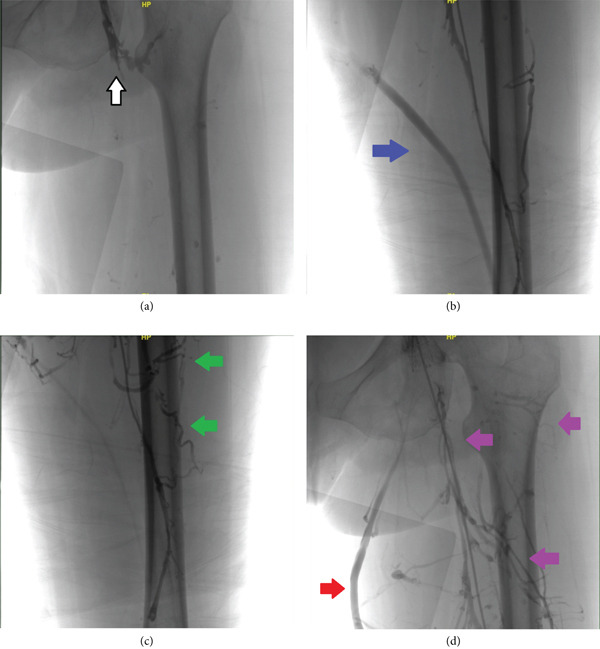
(a) Venography demonstrating complete occlusion at the level of the femoral vein ostium (white arrow). (b) Venography demonstrating enlargement of the left great saphenous vein (GSV) (blue arrow) as a consequence of chronic lower extremity DVT. (c) Venography demonstrating the identification of new collaterals (green arrows) as a sequela of chronic lower extremity DVT. (d) Venography demonstrating enlargement of the left GSV (red arrow) as well as the development of extensive venous collateralization (purple arrows) as a result of recurrent and longstanding DVTs.

At presentation, she was tachycardic, but otherwise hemodynamically stable on room air. Physical exam was notable for diffuse edema and erythema of the left lower extremity. Ultrasound venous duplex of the bilateral lower extremities revealed an occlusive DVT in the left superficial femoral vein with interval improvement from a previous study 5 months prior, which showed DVT in the left superficial femoral vein and left common femoral vein. Computed tomography angiography of the chest showed no evidence of pulmonary embolism. She was started on a heparin drip and interventional cardiology was consulted. She underwent venography that showed a complete occlusion of the left common femoral vein at the ostium with extensive collateral circulation, including a large great saphenous vein supplying the entire venous system of the left lower extremity (Figures 1a, 1b, 1c, and 1d). As a result, she was treated conservatively without further intervention, and no adverse events occurred during her hospitalization. The patient was discharged in a stable condition with continuation of lifelong anticoagulation and planned outpatient follow‐up with her primary care physician, cardiologist, and hematologist for ongoing management.

## 3. Discussion

Diagnosing DVT involves a combination of clinical evaluation, risk stratification, and imaging studies [1]. Ultrasound with Doppler remains the first‐line imaging modality due to its high sensitivity and specificity for detecting proximal DVT. However, in cases of chronic DVT with complex anatomy or suspected venous obstruction, venography remains the gold standard for visualizing venous anatomy, thrombus location, and collateral circulation [3]. In this case, venography revealed a complete occlusion of the left femoral vein with extensive collateralization through the great saphenous vein, guiding conservative management.

Collateral vessel development in cases of chronic DVTs is a compensatory mechanism triggered by venous obstruction. Prolonged thrombotic occlusion impairs venous return to the heart leading to increased venous pressure and venous stasis in the affected limb. The resulting tissue hypoxia and endothelial injury stimulates the release of pro‐angiogenic factors such as vascular endothelial growth factor (VEGF) and basic fibroblast growth factor (bFGF), promoting angiogenesis and vasculogenesis [5]. Preexisting venous channels dilate and form collateral networks to bypass the obstruction, enabling venous return despite impaired primary venous flow. However, these vessels are often structurally inefficient due to the absence of functional valves, predisposing patients to chronic venous insufficiency and PTS [6].

The mainstay of chronic DVT management is continued anticoagulation to reduce the risk of future thrombotic events and pulmonary embolism, particularly when associated with unprovoked DVT or a chronic provoking factor [7]. Additionally, patient education on anticoagulation adherence, regular monitoring, and risk factor modification play a critical role in preventing recurrence and managing symptoms. Compression therapy can aid in symptom relief of venous insufficiency by reducing venous stasis and edema while promoting venous return, though its utility in well‐collateralized cases remains debated [4]. Lifestyle modifications such as increased mobility, weight management, and smoking cessation are also essential adjuncts in conservative management strategies [7].

Mechanical thrombectomy and other invasive therapies—including catheter‐directed thrombolysis, venous stenting, or surgical bypass grafting—have a limited role especially in chronic DVT and are reserved for patients with refractory or severe symptoms, limb‐threatening disease, or marked venous obstruction unresponsive to optimal anticoagulation and compression therapy. Although thrombectomy followed by stenting may be considered for select cases, its efficacy is not established by high‐quality evidence, and available studies demonstrate uncertain benefit for key clinical outcomes, such as PTS, recurrent venous thromboembolism, and quality of life [8]. Contraindications to mechanical thrombectomy include extensive fibrotic organization of the thrombus, high risk of complications such as vessel perforation or bleeding, and poor candidates for invasive procedures with significant comorbidities [8]. When left untreated, chronic DVT leads to fibrotic remodeling, transforming the thrombus into a rigid structure adherent to the vessel wall. This process, known as thrombus organization, results in permanent venous obstruction unamenable to mechanical thrombolysis due to inability of venographic wires and catheters to transverse the hardened thrombus. This scenario not only limits procedural success but also increases procedural risks, such as endothelial injury or iatrogenic thrombosis [9]. Moreover, given the development of effective collateral circulation as seen in the patient, invasive intervention was deemed unnecessary and potentially harmful, which further reinforces the decision for conservative treatment.

The patient′s extensive collateralization, including a hypertrophied great saphenous vein compensating for femoral vein occlusion, is a rare but documented phenomenon. Studies show that well‐formed collaterals may reduce reliance on invasive interventions by partially restoring venous outflow, reducing venous hypertension, and alleviating symptoms supporting the conservative approach adopted in this case [6]. However, limited data exist on long‐term outcomes for such patients, emphasizing the need for ongoing research.

## 4. Conclusion

Understanding the pathophysiology of collateral vessel development and its clinical implications helps refine management decisions, especially in patients at high risk for recurrent thrombotic events. This case highlights the unique adaptive process of collateral formation that allowed conservative management despite extensive venous occlusion. Lifelong anticoagulation remains the cornerstone of management and is paramount in preventing recurrent events. Recognizing adaptive collateral mechanisms can further help avoid unnecessary invasive procedures, thereby reducing morbidity and healthcare costs. This outcome underscores the importance of individualized treatment strategies guided by venographic findings, clinical presentation, and patient‐specific risk factors such as thrombophilia. Future research should focus on long‐term outcomes in patients with extensive venous collaterals, evaluating prognostic factors and potential improvements in management protocols.

**Nomenclature**
bFGF: basic fibroblast growth factor
DVT: deep venous thrombosis
PTS: post‐thrombotic syndrome
VEGF: vascular endothelial growth factor

## Ethics Statement

Institutional ethical review was not required for a single‐patient case report.

## Consent

Prior to development and submission of the manuscript, written informed consent was obtained from the patient, including accompanying images.

## Conflicts of Interest

The authors declare no conflicts of interest.

## Author Contributions

Demi Curbelo, MD: writing—original draft preparation (lead), conceptualization (lead). Ian Lancaster, MD: conceptualization (supporting), writing—reviewing and editing (equal). Alexander Yaylayan, MD: writing—reviewing and editing (equal). Merrill Krolick, DO: supervision (lead).

## Funding

No funding was received for this manuscript.

## Data Availability

No datasets were generated or analyzed during this study. As a case report, the patient′s individual information is not available for review, in accordance with HIPPA compliance.
